# A dual-band reflective polarizer based meta-surface with higher angular stability for C and X-band applications

**DOI:** 10.1038/s41598-024-80734-2

**Published:** 2024-11-26

**Authors:** Saranya Madheswaran, Rajeshkumar Venkatesan

**Affiliations:** Department of Communication Engineering, School of Electronics Engineering, Vellore Institute of Technology, Vellore, Tamil Nadu India

**Keywords:** Axial ratio, Linear-to-circular polarization, Polarization conversion ratio, Reflective type polarizer, Satellite communication, Engineering, Electrical and electronic engineering

## Abstract

A reflective dual-band linear-to-circular polarizer for C and X-band application is presented. The proposed elliptical Frequency Selective Surface (FSS) is capable of enhancing the polarization control and minimizes the cross-polarization compared to traditional circular or square geometries. The linear-to-circular polarization is achieved through two elliptical apertures, where each shape generates orthogonal modes with a 90° phase shift. The inner element targets higher frequencies while the outer element handles lower frequencies, enabling efficient polarization conversion across both bands. The proposed unit cell comprises 14 mm × 14 mm and is fabricated on Rogers RT5880 substrate. It exhibits a reflective polarizer at a center frequency of 7.70 GHz with a bandwidth of (7.64–7.76 GHz) and other at 9.42 GHz with a bandwidth of (9.27–9.57 GHz), respectively. In the proposed FSS, the 7.70 GHz exhibits LH-CP, and RH-CP conversion is provided at 9.42 GHz. In addition, an axial ratio of the FSS of (< 3 dB) demonstrated a circular polarization with a polarization conversion ratio (PCR) of 99.9%. In addition, an equivalent circuit model (ECM) of a reflective polarizer has been verified. Furthermore, angular stability of the FSS can be achieved at various incident angles at 0°, 30°, 60°, and 90°. The polarization conversion operates in frequency bands of C-/X, suitable for wireless systems, radar systems, electromagnetic shielding, and satellite communications.

## Introduction

Circular polarization (CP) plays an important role in various applications utilizing frequency-selective surfaces. Polarizers are one of the applications of Frequency-Selective Surfaces (FSS). FSS can be designed efficiently to convert linearly polarized (LP) waves into circularly polarized (CP) waves or vice versa. It is used to filter out unwanted waves, ensuring that only the desired circular polarized wave is transmitted or received. Compared to linear polarization, circular polarization signals are less susceptible to multi-path fading caused by reflections from objects. This leads to improvement in the signal quality and robustness, particularly in challenging environments^1^. Furthermore, improved signal penetration in some cases, CP signals can penetrate obstacles better than linear polarization. In the microwave system, circularly polarized waves are suitable for applications like polarization control of THz communication systems, sensing and imaging^2^, RFID to improve tag detection and target identification, radar applications, RCS reduction^3^, satellite communication^4^, radomes^5^. In satellite earth link communication systems, the LP wave causes unexpected fluctuations while passing through the ionosphere. This rotation may result in a polarization mismatch at the receiver that will impact the link budget systems. Due to the simplicity of switching between up-link and down-link polarization sensing, circularly polarized (CP) signals are frequently employed in wireless communications specifically satellite communications^6^. In particular, the current trend in satellite communications is to shift onto higher frequencies and integrate the broadcast and receive antennas into a single dual-band terminal. However, because of the conflicting objectives, the component design and production are quite challenging. Alternatively, circular polarization waves have been used for the specified applications stated to increase the propagation link budget and polarization efficiency^7^. In comparison to linear polarized waves, circular polarized waves provide excellent capacity against polarization mismatch and multi-path fading. Furthermore, to enhance the isolation between transmit and receive signals, an antenna for satellite communications usually required to operate on two different and non-adjacent frequency bands in orthogonal polarizations^8^. Generally, polarization converters are used to convert transmission polarization and reflection polarization. A slab of an anisotropic medium known as a polarization converter is used to change the polarization of an incident wave from one type of wave, such as linear to circular or vertical to horizontal. Depending on the direction of polarization rotation there are two types of polarization converters: transmission type and reflection type. The most common methods for implementing planar polarization converters are frequency-selective surfaces or other kinds of periodic structures. An isotropic 2-D FSS is typically used in the design of reflective polarization converters widely used in reflecting array antennas and radar stealth applications^9^. Depends on the phase difference of $\frac{{\uppi }}{2}$ or $- \frac{{\uppi }}{2}$ a polarization mode of CP wave, it operates in either left-handed circular polarization (LH-CP) or right-handed circular polarization (RH-CP).

Several polarization conversions have been discussed in the literature during the last few decades. A dual-band linear polarization to circular polarization^10,11^ is suitable for X-/K/Ka-band satellite applications. A wideband cross junction FSS has a center frequency at 6 GHz with a fractional bandwidth of 9.6% illustrated^12^. An orthogonal polarization converter^13^ that exhibits a dual-band resonant frequency of circular polarization wave has been presented. In a cross-shaped^14^ composite FSS of broadband circular polarization achieved 74% bandwidth from 5.15 to 11.20 GHz and different incident angles ranging from 0° to 20° for both TE and TM modes were analysed to determine the angular stability. A multi-layer dual-band transmission type linear to circular polarization is presented^15^ provides a resonant frequency of 6.4–8.8 GHz and 12.1–13.9 GHz which operates less than 3 dB axial ratio with fractional bandwidth of 31.6% and 13.8%. The angular stability of incident angles 0°, 15°, and 25° are analysed. A Meandered split ring^16^ structure illustrated single band linear to circular polarization at resonant frequency of 3.5 GHz and angular stability of various oblique incidence from 0° to 30° is measured. A broad-band transmission FSS consists of a circular ring resonator and a split square ring^17,18^ achieved LP-to-CP. Other than the structures mentioned above, two-dimensional arrays of the split ring resonator^19–21^, Jerusalem cross^22–24^ double square loop^25^, meandered line^26^ symmetric cross-shaped^27^ V-shaped dipole elements and slots, fish-shaped slot^28,29^ were reported. A reflective polarization^30^ at the frequency range of 4.40–5.30 GHz, 9.45–13.60 GHz and PCR efficiency of 86% has been presented. The different FSS geometry of LP-to-CP conversion for terahertz metamaterial, C/X band, and RCS applications are discussed and angular stability is stable up to 45°^31–33^. From the existing literature, a dual-band polarizer with transmission and reflection type of polarization converter is discussed.

In the proposed FSS, a dual-band reflective type polarization conversion is achieved with a frequency range from 4 to 12 GHz suitable for various applications like wireless systems, radar, and satellite communication. Orthogonal modes with a 90° phase shift are provided by elliptical shape FSS. It improves polarization control, filtering and reduce interference, making it suitable for applications that require robust dual-band performance with reliable LP-to-CP conversion. The left-handed and right-handed circular polarization was observed using surface current distribution at the operating frequency. Angular stability of different incident angles from 0° to 90° has been analyzed with axial ratio, reflection coefficient, and Polarization Conversion Ratio.

The content of the present work is given as follows: “Proposed unit cell design” section deals with the design and analysis of the proposed FSS, whereas “Angular stability of proposed FSS” section presents the angular stability analysis followed by a parametric study of unit cells in “Parametric study” section. In “Validation of proposed FSS” section discusses the performance and validation through surface current distribution of RHCP, LHCP, and equivalent circuit model. Hardware fabrication and measurement results are presented in “Experimental results and validation” section and the proposed work is summarized in “Conclusion” section.

## Proposed unit cell design

**Fig. 1:**
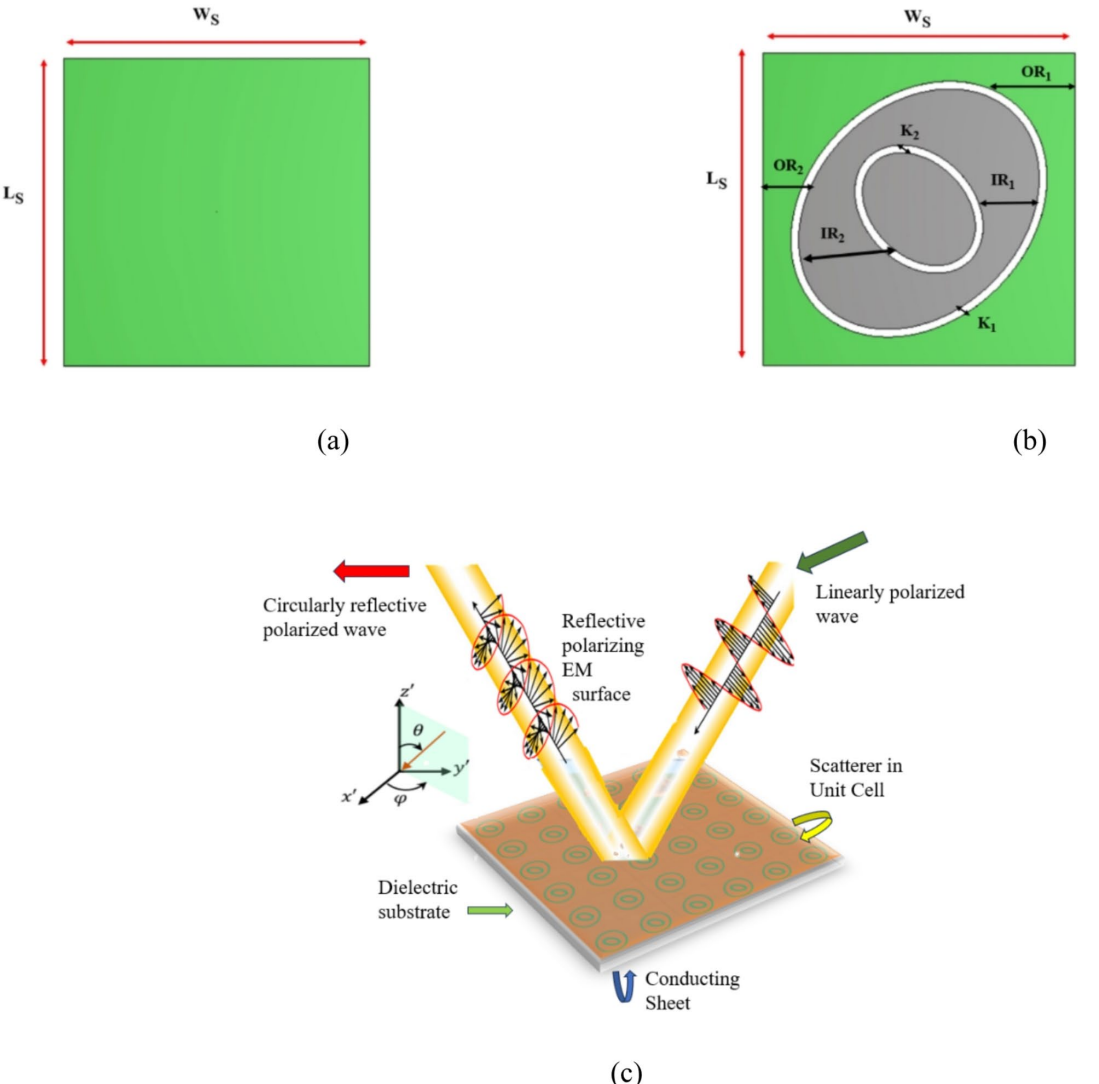
Reflective polarizer of proposed FSS (**a**) Ground plane (**b**) Top view (**c**) LP-to-CP conversion.

The proposed FSS consists of an elliptical shape with inner and outer elements and the bottom view of a metallic ground plane is shown in Fig. 1a. In the top view, a unit cell composed of dual elliptical-shaped elements which is etched on Rogers RT5880 substrate with a relative permittivity of 2.2, and loss tangent of 0.0009. The dimension of the proposed unit cell is 14 mm × 14 mm, h = 1.6 mm, and copper conductive layer thickness of 0.035 mm which is shown in Fig. 1b. Elliptical structures in FSS offer several novelties and advantages compared to traditional circular or square geometries. It can be designed to exhibit specific polarization properties, such as circular polarization or polarization filtering. It minimizes the cross-polarization which is essential for applications requiring high polarization purity. Ensure the unit cells are symmetrical to minimize cross-polarization effects. Polarization conversion is achieved by controlling the geometry and arrangement of the FSS elements. To achieve LP-to-CP conversion the FSS elements can be designed to introduce a phase shift between the horizontal and vertical components of a linearly polarized wave. The wave becomes circular polarized, when the phase shift is exactly 90°. This phase shift can be used to selectively reflect or transmit waves of a specific polarization. The phase shift will lead to the polarization conversion. A linearly polarized wave is incident on the FSS is converted to circular polarization by introducing the phase shift of a linearly polarized wave. Figure 1c shows a linear-to-reflective circular polarization conversion. Circular polarization is achieved by a reflected wave due to the phase-shift of exactly 90° between horizontal and vertical reflected elements. The circular polarization achieved (LHCP) and (RHCP) of reflected wave with the same performance of the FSS. The dimensions of the unit cells are shown in Table 1. When an electromagnetic wave interacts with FSS, it excites various resonant modes depending on its polarization and frequency. Based on the geometry of FSS and material properties, resonances interact with different polarizations leading to selective transmission or reflection type. The proposed meta-surface was executed by using CST microwave studio software. To compute the reflection and transmission coefficients a frequency domain solver has been utilized for analysing the floquet ports and boundary conditions of x and y directions. A linearly polarized incident wave is excited through an FSS layer an outer elliptical element that is tilted with 45° in the x-y plane and z-direction for extracting the scattering coefficient. The electric field consists of vertical and horizontal elements. During the propagation of incident wave to the FSS elements, generates a phase difference of 90°. Hence, a reflective circular polarization has been achieved.

**Table 1:** Physical dimensions (in mm) of the proposed FSS.

Parameters	L_s_ = W_s_	OR_1_	OR_2_	IR_1_	IR_2_	K_1_ = K_2_	h	Angle rotation (degree)
Values in mm	14	6.4	5.0	3.25	2.45	0.35	1.6	ROT_1_—45° (outer element)
								ROT_2_—135° (inner element)

L_s_ = W_s_—substrate length and width, OR_1_, OR_2_—outer element radius, IR_1_, IR_2_—inner element radius, K_1_ = K_2_—gap between inner and outer element.

**Fig. 2:**
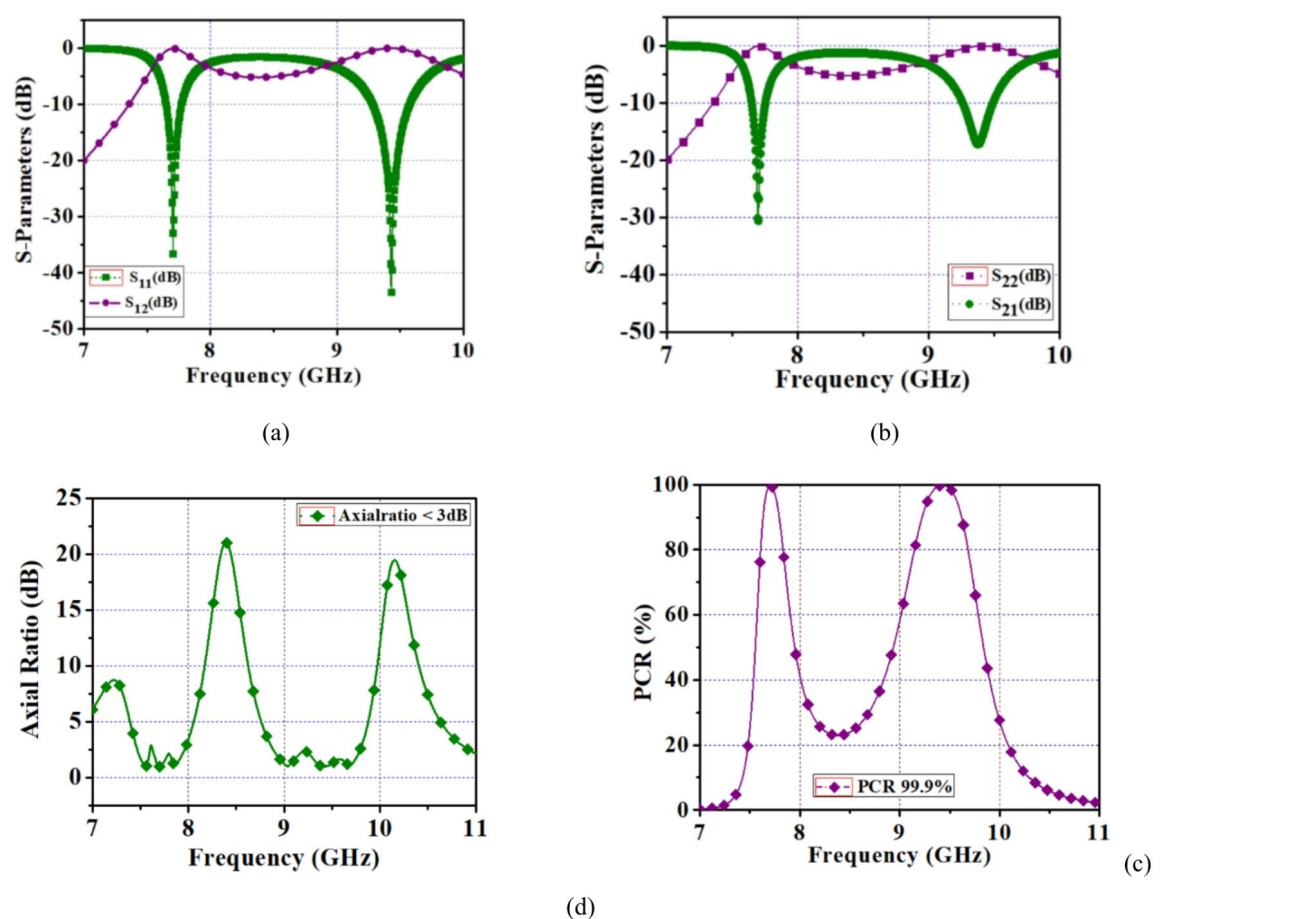
(**a**) Proposed reflection coefficient (TE mode), (**b**) TM mode, (**c**) Axial ratio, (**d**) PCR.

In addition, a reflective wave generates a circular polarization, when the field elements are orthogonal to other elements with equal amplitudes. Due to a symmetric structure of FSS TE and TM modes generated the same resonant frequency. The reflective type (TE-mode) and (TM-mode) are shown in Fig. 2a and b. To know the effectiveness of LP-to-CP conversion a polarization conversion ratio (PCR) is calculated.

### Polarization conversion ratio

PCR is a measure of how efficiently a FSS can convert LP-to-CP waves. It can be computed by using the transmission coefficients for the co-polarized and cross-polarized components. A high PCR indicates that the FSS is effectively converting LP-to-CP with minimal cross-polarization. The polarization conversion ratio^34^ is computed by using Eq. (1).

1 $${\text{PCR}} = \frac{{|r_{xy} |^{2} }}{{|r_{xy} |^{2} + |r_{yy} |^{2} }}$$

where r_xx_, r_yy_—co-polarized coefficients, r_yx_, r_xy_—cross-polarized coefficients. The co-polarization coefficient of r_yy_, r_xx_ is expressed in Eq. (2).

2 $${\text{r}}_{{{\text{xx}}}} = |{\text{r}}_{{{\text{xx}}}} |e^{{\upphi_{xx} }} ,\quad {\text{r}}_{{{\text{yy}}}} = |{\text{r}}_{{{\text{yy}}}} | e^{{\upphi_{yy} }}$$

When a cross-polarized reflection co-efficient $R_{xy} = 1$ at the time E-field elements are transmitted properly without any reduction in signal strength within a specified frequency. A phase difference among the two reflective components is given as ∆ϕ = ϕ_xx_ − ϕ_yy_. The reflective wave generates cross-polarization must satisfy the condition of ∆ϕ =  ± 180°, and |r_xx_| = 0, |r_xy_| = 1. When ∆ϕ provides other values except ± 90°, ± 180° an elliptical reflective polarization can be obtained. Furthermore, to generate a circular reflective type polarization reflection coefficient of phases and magnitudes needs to satisfy the following conditions given in Eq. (3).

3 $$\left| {{\text{r}}_{{{\text{xx}}}} } \right| = \left| {{\text{r}}_{{{\text{yy}}}} } \right|\;\;{\text{and}}\;\;\Delta\upphi = \pm 90^\circ$$

In proposed FSS, by using Eq. (1) the polarization conversion ratio of 99.9% has been achieved.

### Axial ratio

The axial ratio is an essential parameter to achieve a circular polarization and determine an E-field rotation of reflected waves. The axial ratio is computed by using the following expression,

4 $${\text{Axial ratio}} = \left( {\frac{{|R_{xx} |^{2} + |R_{xy} |^{2} + \sqrt c }}{{|R_{xx} |^{2} + |R_{xy} |^{2} - \sqrt c }}} \right)^{\frac{1}{2}}$$

where c = |*R*_*xx*_|^4^ + |*R*_*xy*_|^4^ + 2|*R*_*xx*_|^2^ |*R*_*xy*_|^2^ cos(2Δϕ).

By using Eq. (4) an axial ratio of the proposed unit cell is computed as less than 3 dB (AR < 3 dB) therefore it achieved a reflective circular polarization which is shown in Fig. 2c.

From the proposed FSS, a reflective polarizer achieved a dual-band circular polarization within a C-/X-band. Also, a performance of polarization conversion ratio of 99.9% is computed as shown in Fig. 2d. The left-handed and right-handed circular polarizations are observed from the surface current distribution.

## Angular stability of proposed FSS

In general, angular stability is observed based on the geometry of FSS and the spacing of an inter-element distance. An incoming wave could never be perpendicular to the polarization conversion plane. Due to the type of antenna radiation or different sources angular stability can be deflected or oblique. Hence, oblique angles of various incident angle performances of LP to CP conversion should be studied. A large inter-element separation provides multiple lobes, and very small inter-element spacing maintains a constant resonant frequency at various incident angles. In addition, while increasing an inter-element spacing consistently, it is capable of improving an oblique stability of the FSS’s. In the proposed FSS, a separation of an inter-element spacing is small, it achieved the same resonant frequency for different angles of incidence^35^.

**Fig. 3:**
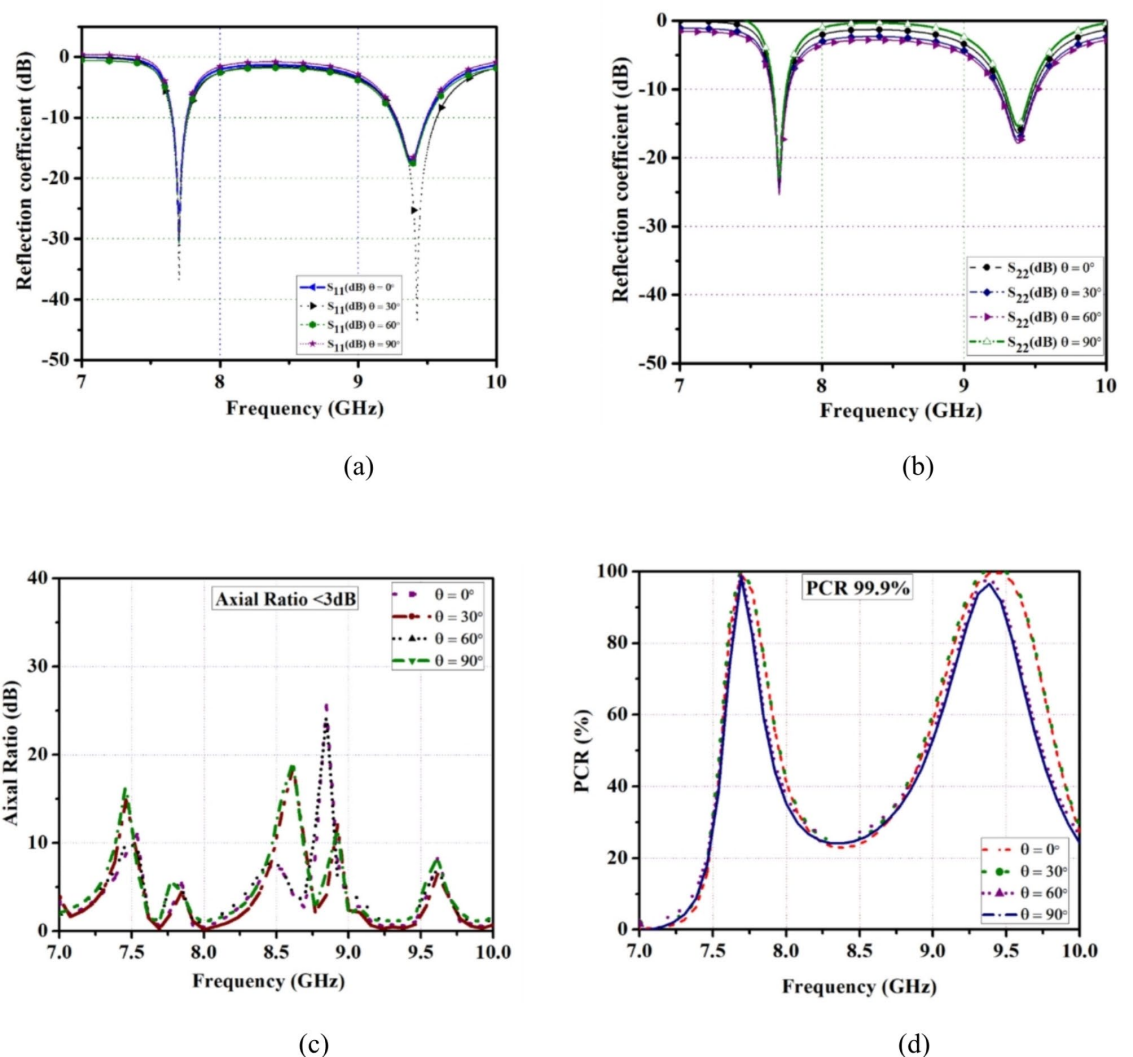
Angular stability: (**a**) TE mode, (**b**) TM mode (**c**) Axial ratio (**d**) PCR.

In proposed FSS, a reflective circular polarization of TE and TM mode exhibits an oblique angle at different incident angles from θ = 0° to 90° as shown in Fig. 3a and b. Figure 3c. It illustrates the axial ratio (AR < 3 dB) maintains a constant value for various incident angles up to 90°. Also, the polarization conversion ratio gives a conversion efficiency of 99.9%, and remains the same result of oblique angles as shown in Fig. 3d. It is noted from the proposed unit cell, it maintains the same center frequency of various incident angles from θ = 0° to 90°. It shows that the proposed FSS has excellent angular stability.

## Parametric study

**Fig. 4:**
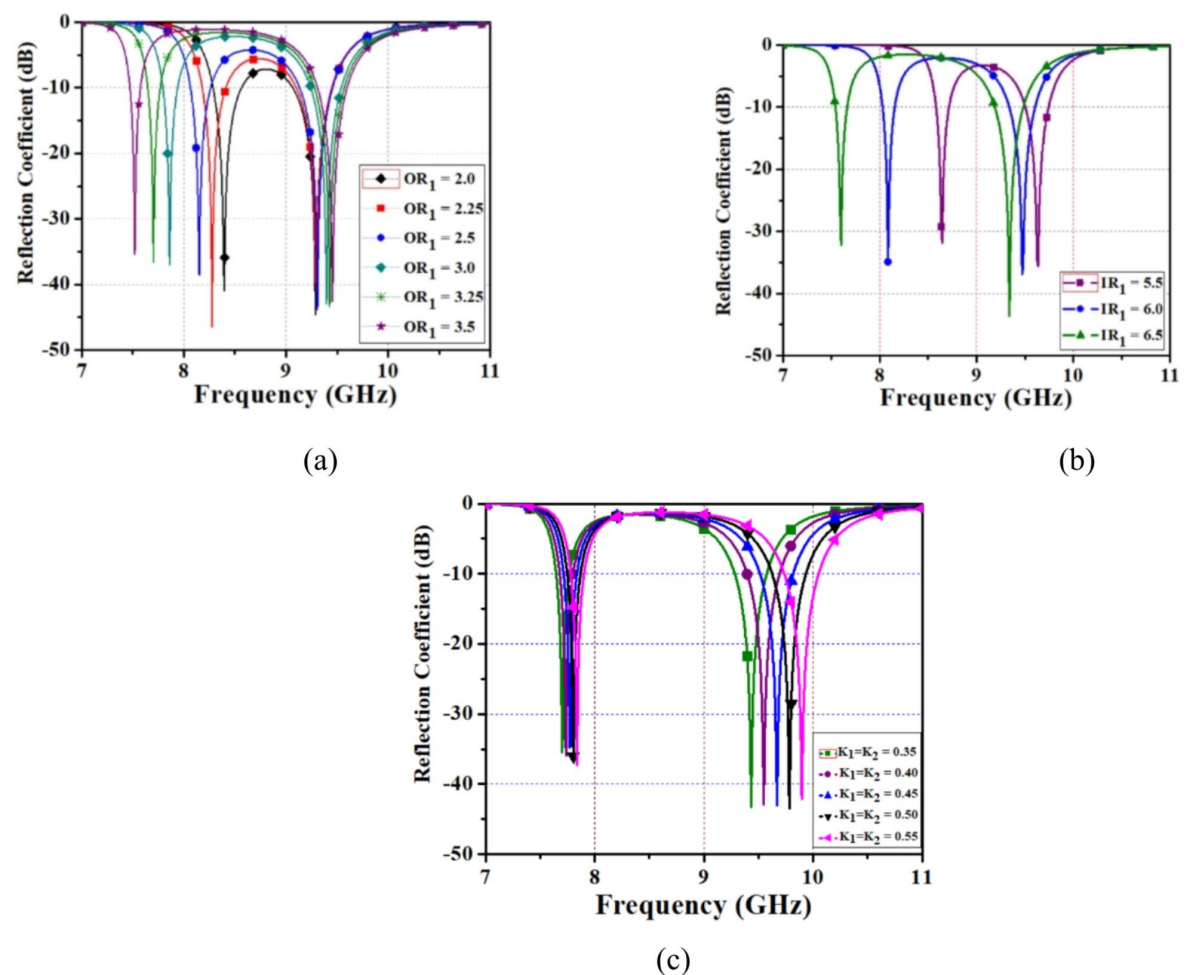
Parametric analysis of FSS (**a**) variation of outer-element width (**b**) variation of inner-element width, (**c**) Variation of K_1_ and K_2_.

The parametric analysis is a critical tool for understanding and optimizing the performance of Frequency Selective Surfaces. By systematically varying specific parameters of the FSS such as unit cell dimensions, substrate thickness, and operating frequency different parametric analysis has been simulated. By optimizing these parameters, FSS can able to enhance the performance at different resonant frequency, bandwidth, polarization response, polarization conversion (LP-to-CP), and angular stability. The resonant frequency of the FSS is highly sensitive to the dimensions of the unit cells. Even small changes in size can significantly affect the operating frequency due to the changes in equivalent inductance and capacitance of the unit cell. A parametric study was simulated by changing the inner and outer element width of the proposed unit cell. From the parametric analysis varying the inner-element, outer-element width and inter-spacing distance of the unit cell produces a different resonant frequency, and relative bandwidth was analysed. By changing the outer-element width of a unit cell from (OR_1_ = 2.0 mm to 3.5 mm) dual-bandwidth and centre frequency is observed. From 2.0 to 2.5 mm, the bandwidth of the center frequency is low when compared to other values as shown in Fig. 4a. In addition, varying inner-element width of (IR_1_ = 5.5 mm to 6.5 mm) dual resonant frequency is noted as shown in Fig. 4b. A variation of an inter-element spacing distance K_1_ and K_2_ from (0.35 mm to 0.55 mm) gives a dual frequency and bandwidth also analyzed as shown in Fig. 4c. As a result, during parametric studies the modification of the unit cell, gives a slight variation in center frequency and bandwidth.

Finally, based on the performance of different parameter values desired centre frequency and bandwidth are chosen. Therefore, from the parametric study, a resonant frequency of 7.70 GHz and 9.40 GHz is obtained at OR_1_ = 3.5 mm, IR_1_ = 6.5 mm, and K_1_ = K_2_ = 0.35 mm. It provides a better bandwidth and maintains angular stability of unit cell from θ = 0° to 90° while compared to other values and suitable for C-/X-band applications.

## Validation of proposed FSS

### Surface current distribution

Incoming electromagnetic waves interact with a metasurface; it generates electric and magnetic dipole moments resulting in meta-atoms become polarized. Due to the coupling between these dipoles, it produces multiple electric and magnetic resonances which enhance a bandwidth of the polarization. By computing the effective permeability and permittivity, surface impedance can be calculated. The surface impedance controls the flow of surface current because the current continuously searches a low-impedance path. The surface impedance is computed by using the given equation, ${\upeta }_{{\text{s}}} = \sqrt {\frac{{{\upmu }_{{{\text{eff}}}} }}{{{\upvarepsilon }_{{\text{eff}}} }}}$.

**Fig. 5:**
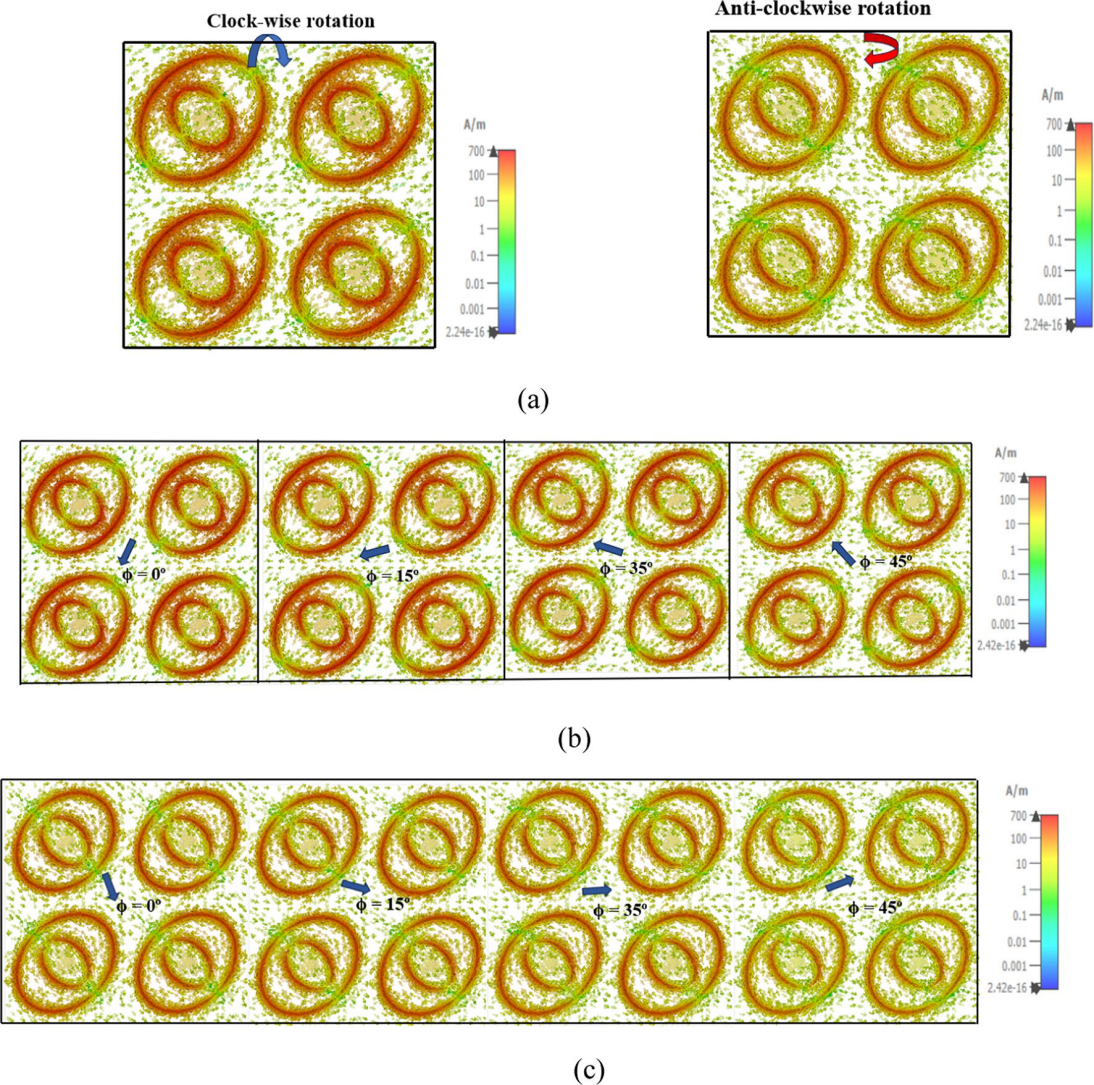

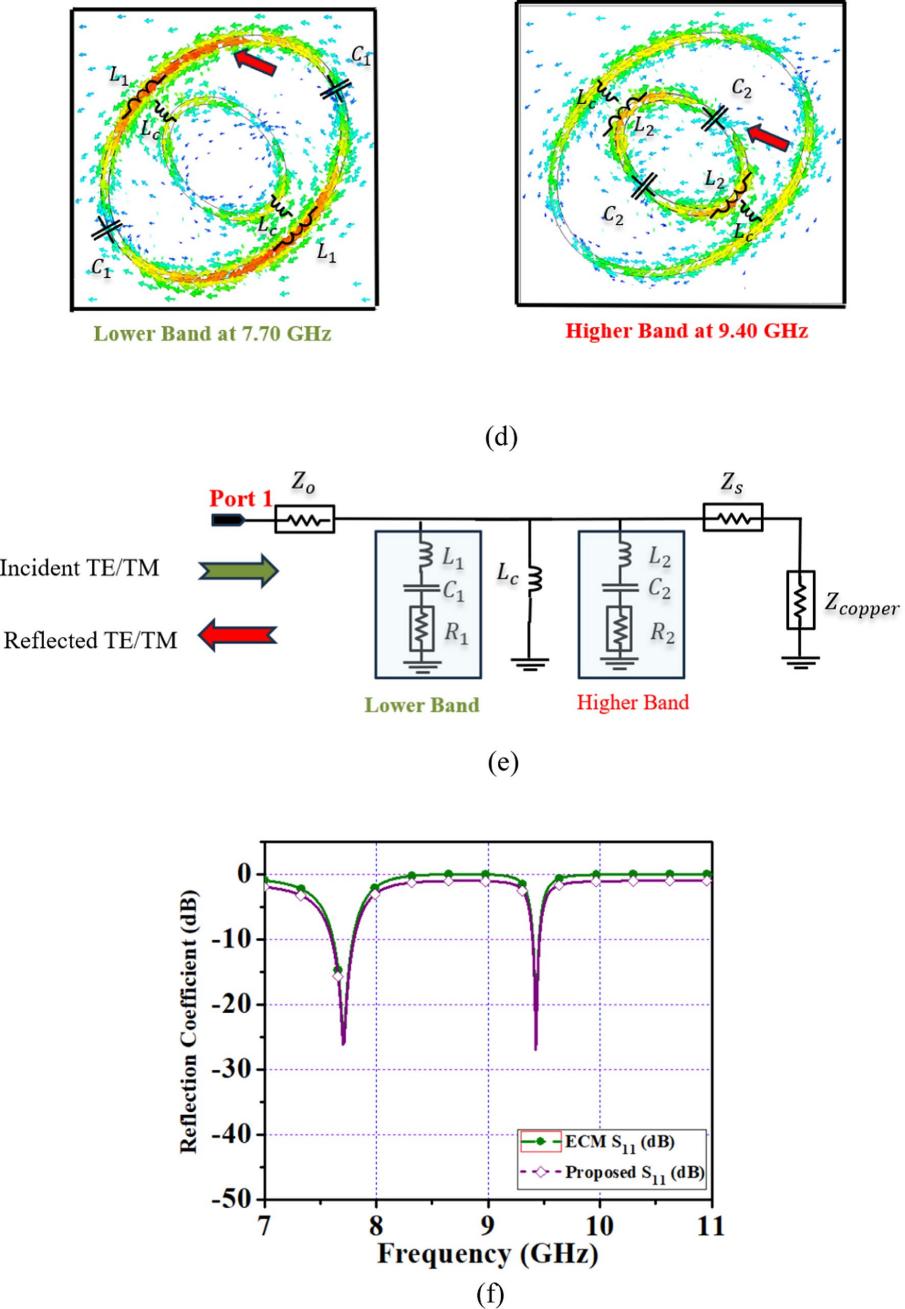
Surface current: (**a**) LHCP, RHCP (**b**) ϕ = 0°, 15° 35° to 45° (LHCP) (**c**) ϕ = 0°, 15° 35° to 45° (RHCP) (**d**) lumped elements of proposed FSS, (**e**) ECM, (**f**) S_11_ (dB).

The surface current distribution is verified for the resonant frequency of 7.70 GHz and 9.42 GHz. It is observed that from the surface current, a circular polarization was attained. At 7.70 GHz the current distribution exhibits a left-handed circular polarization (clockwise rotation) and at 9.42 GHz generates a right-handed circular polarization (anti-clockwise) as shown in Fig. 5a. In surface current distribution, by changing the different phase angles, LHCP and RHCP has been verified with different polarization azimuth angles at ϕ = 0°, 15° 35° to 45° as shown in Fig. 5b and c. Hence, the proposed FSS achieved a dual-band reflective circular polarization, and according to the surface current LH-CP, RH-CP has been observed at the center frequency.

### Equivalent circuit model of FSS

A dual-band FSS equivalent circuit model for linear-to-circular polarization of transmissive and reflective types are discussed^36–38^. To validate the performance of a dual-band reflective polarizer an equivalent circuit model has been designed. The geometry of the proposed FSS consists of the inner and outer elliptical elements. Figure 5d illustrates the lumped elements of a unit cell. An outer element that gives a lower-band center frequency of 7.70 GHz, it consists of L_1_, C_1_ and L_c_ elements. Similarly, inner elements are L_2_, C_2_, and L_c_ that exhibits a higher-band resonant frequency of 9.42 GHz.

A reflective polarizer of a dual-band FSS is validated with ECM which is shown in Fig. 5e. The circuit modeling and optimization of circuit parameters are carried out using the CST schematic tool. The lower band, is contributed by series R_1_, L_1_ and C_1_ whereas the higher band is due to R_2_, L_2_, and C_2_. The coupling between an inner and outer element is represented by the coupling inductor L_c_. The impedance is dominated by the dielectric materials as denoted by Z_s_, while the free space impedance is represented by Z_η_. The bottom view completely covered by copper which is represented by Z_c_. The lumped element values are given L_1_ = 0.218 nH, C_1_ = 1.033 pF, R_1_ = 0.0429 Ω, L_2_ = 0.900 nH, C_2_ = 0.363 pF, R_2_ = 0.0560 Ω, Z_η_ = 377 Ω, Z_s_ = 39.805 Ω, Z_c_ = 18.30 Ω. An equivalent circuit model is a series resonance circuit. In general resonance frequency is given as,

5 $${\text{f}}_{{\text{r}}} = \frac{1}{{2 \pi \sqrt {LC} }}$$

The impedance of the resonant circuit is computed by

6 $${\text{Z}} = \sqrt {{\text{R}}^{2} + {\text{j}}\left( {{\text{X}}_{{\text{L}}} - {\text{X}}_{{\text{C}}} } \right)^{2} }$$

By using f_r_ condition an impedance in series RLC circuit is always equal to capacitance X_L_ = X_C_. In order to satisfy a resonant condition, the circuit must be purely resistive, and imaginary part of the impedance is equal to zero. Therefore, an impedance of the circuit is given in Eq. (7).

7 $${\text{Z}} = \sqrt {{\text{R}}^{2} + 0^{2}} ,\;\;{\text{Z}} = {\text{R}}$$

The RLC parameters of the proposed FSS are optimized and compared with EM simulation as shown in Fig. 5f.

It is noted that from the ECM analysis, a dual-band reflective polarizer produces the same center frequency of 7.70 GHz and 9.42 GHz, agree well with the simulation.

## Experimental results and validation

**Fig. 6:**
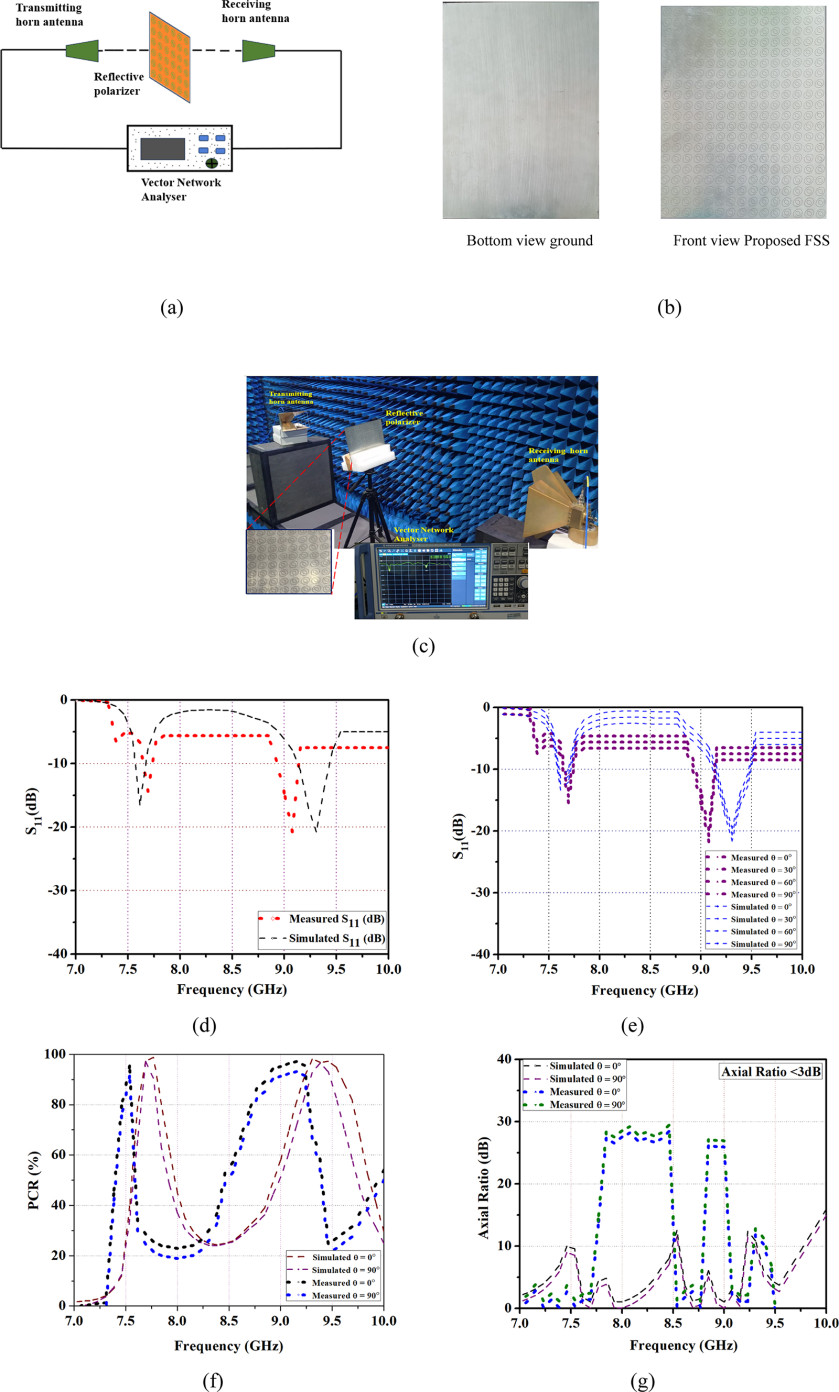
Measured and simulated results (**a**) Block diagram (**b**) Fabricated prototype (**c**) Measurement setup (**d**) S_11_ (dB), (**e**) S_11_ (dB) angular stability (0°–90°), (**f**) PCR, (g) Axial ratio.

The Proposed FSS of the reflective type polarizer has been fabricated on a Rogers RT5880 substrate with a thickness of 1.57 mm. A transmitting and receiving horn antenna are coupled with a vector network analyser to observe the reflection, transmission coefficient, and angular stability of FSS. A schematic of the measurement setup is shown in Fig. 6a. The reflective polarizer of an array of elements consists of 20 × 20-unit cells with a total dimension of 280 × 280 mm^2^ is fabricated and shown in Fig. 6b.

To validate the performance of a reflective polarizer, it is measured in an anechoic chamber as shown in Fig. 6c. A fabricated FSS has been placed on a rotating surface with a far-field distance from standard transmitting and receiving horn antenna. The reflective polarizer can be placed at a 2 m distance from the panel. To transmit or receive x- and y-polarized waves the horn antenna is placed on longer and shorter sides of the prototype. By rotating a spiral arm of a receiving antenna to receive the transmitted or reflected wave while a transmitting antenna is fixed perpendicular to the polarizer. The measurements can be observed in two ways one is without a sample and the other way is after placing a sample. From the measured result, a reflection coefficient and angular stability are noted. The measurement result provides a better degree of agreement with simulation and ECM simulation. This convergence between empirical measurements and computational predictions validates our FSS design and underscores its effectiveness in achieving the desired reflective polarizer for C-/X-band applications.

From the measured results, a dual-band reflective polarizer gives a center frequency of 7.5 GHz and 9.2 GHz. Angular stability is maintained up to 90°. It is noted that the measured results of reflection coefficients are matched with the simulation results, along with a slight variation which is shown in Fig. 6d. The variation is due to the losses caused by the substrate and tolerance. Due to the symmetric FSS structure, both TE and TM modes are equal. Figure 6e shows angular stability of the proposed FSS for various incident angles at θ = 0°, 30°, 60°, 90° and maintains a constant resonant frequency. The performance of polarization conversion from LP-to-CP is formulated by PCR. It shows the effectiveness of the conversion of proposed the unit cell. A polarization conversion ratio achieved an excellent result of 99.9% as shown in Fig. 6f. Figure 6g illustrates the axial ratio which exhibits AR < 3 dB a linear-to-circular polarization of the FSS is achieved. Similarly, angular stability of an axial ratio for θ = 0° is noted and it maintains the same result for various incidences up to 90°. Finally, the proposed FSS covers a resonant frequency of (C-/X-band) and also achieved better angular stability of incident angles at θ = 0°, 30°, 90° for all the parameters. A dual-band linear-to-circular polarization reflective type is suitable for satellite applications, radar, shielding, and wireless communication. The performance of a proposed FSS is compared with the literature listed in Table 2. From the comparison table, it is noted that the proposed FSS achieved reflection co-efficient, LHCP, RHCP, PCR, and axial ratio also provides a better angular stability up to 90°.

**Table 2:** Comparison of proposed FSS with literature.

Refs.	Centre frequency (GHz)	Substrate/dielectric constant	No. of layers	Size of unit cell (λ_0_)	LHCP/RHCP	PCR (%)	AR (dB) or AR bandwidth (%)	Angular Stability
^8^	4.40–5.30 GHz and 9.45–13.60 GHz	FR4/4.4	2	0.22λ_0_ × 0.22λ_0_	–	86%	–	-
^9^	9.65 GHz and 10.57 GHz	RT5880/2.2	2	0.35 λ_0_ × 0.35 λ_0_	–	–	8%	0°–30°
^11^	7.5–7.7 GHz and 11.5–11.9 GHz	FR-4/4.4	2	0.26 λ_0_ × 0.26 λ_0_	–	95%	< 3 dB	0°–45°
^14^	5.15 to 11.2 GHz	RT5880/2.2	4	0.25 λ_0_ × 0.25 λ_0_	LHCP/RHCP	> 90%	< 3 dB	0°–20°
^15^	6.4–8.8 GHz and 12.1–13.9 GHz	RT5880/2.2	4	0.22λ_0_ × 0.23λ_0_	–	67.7%	31.6% 13%	0°–25°
^24^	6.1 GHz to 12.6 GHz	F4B-2/2.65	2	0.19 λ_0_ × 0.19 λ_0_	–	–	69%	0°–30°
^27^	2.4 GHz and 5.8 GHz	FR-4/4.4	1	0.26 λ_0_ × 0.26 λ_0_	LHCP/RHCP	–	15.8% 12.6%	0°–45°
^32^	5.74–7 GHz 8.63–10.15 GHz	FR-4/4.3	2	0.21λ_0_ × 0.21λ_0_	LHCP/RHCP	> 90%	< 3 dB	0°–45°
^33^	19.55 − 24.8 GHz 20*.*24 GHz and 23*.*66 GHz	FR-4/4.3	2	0.16λ_0_ × 0.16λ_0_	LHCP/RHCP	99%	< 3 dB	0°–45°
^34^	2.45–2.69 GHz and 7.56–8.1 GHz	FR-4/4.4	1	0.35 λ_0_ × 0.35 λ_0_	LHCP/RHCP	> 90%	< 3 dB	0°–40°
Proposed FSS	7.70 GHz and 9.42 GHz	RT5880/2.2	2	0.35 λ_0_ × 0.35 λ_0_	LHCP/RHCP	99.9%	< 3 dB	0°–90°

## Conclusion

This work presents a polarization conversion of LP-to-CP depending on reflective type FSS which exhibits a dual-band in the frequency range of 4–12 GHz. The reflective polarizer achieved a center frequency of 7.70 GHz in the lower band and 9.42 GHz in the higher band. LP-to-CP conversion has been achieved by 90° phase shift from elliptical FSS and axial ratio (AR < 3 dB). The LHCP at (7.70 GHz) and RHCP of (9.42 GHz) is observed from the surface current distribution and verified with various azimuth angles from ϕ = 0° to 45°. The polarization conversion ratio of the proposed FSS’s has been achieved up to 99.9%. From the results, a proposed unit cell exhibited higher angular stability of different angles of the incident from θ = 0°–90° for reflection co-efficient, axial ratio and polarization conversion ratio. In addition, simulated results of the FSS’s are validated with measured results and an equivalent circuit model. The proposed FSS is suitable for applications including wireless communication, shielding, point-to-multipoint communications, polarimeters, and satellite communications.

## Data Availability

The datasets used and/or analysed during the current study available from the corresponding author on reasonable request.
